# The Combination of Fasting, Acute Resistance Exercise, and Protein Ingestion Led to Different Responses of Autophagy Markers in Gastrocnemius and Liver Samples

**DOI:** 10.3390/nu12030641

**Published:** 2020-02-28

**Authors:** Ana P. Pinto, Tales S. Vieira, Bruno B. Marafon, Gabriela Batitucci, Elisa M. B. Cabrera, Alisson L. da Rocha, Eike B. Kohama, Kellen C. C. Rodrigues, Leandro P. de Moura, José R. Pauli, Dennys E. Cintra, Eduardo R. Ropelle, Ellen C. de Freitas, Adelino S. R. da Silva

**Affiliations:** 1 Postgraduate Program in Rehabilitation and Functional Performance, Ribeirão Preto Medical School, University of São Paulo (USP), Ribeirão Preto, São Paulo 14049-900, Brazil; anapp_5@usp.br (A.P.P.); alisson.rocha@usp.br (A.L.d.R.); eike.kohama@usp.br (E.B.K.); 2 Postgraduate Program in Nutritional Science, State University of São Paulo Júlio de Mesquita Filho (Araraquara). Araraquara, São Paulo 14800-903, Brazil; talessv@hotmail.com (T.S.V.); gabibatitucci@gmail.com (G.B.); ellenfreitas@usp.br (E.C.d.F.); 3 School of Physical Education and Sport of Ribeirão Preto, University of São Paulo (USP), Ribeirão Preto, São Paulo 14040-907, Brazil; bruno.marafon@usp.br; 4 Institute of Translational Nutrigenetics and Nutrigenomics, Department of Molecular Biology and Genomics, Health Sciences University Center, University of Guadalajara, Guadalajara 44100, Mexico; eli_embc@hotmail.com; 5 Laboratory of Molecular Biology of Exercise (LaBMEx), School of Applied Sciences, University of Campinas (UNICAMP), Limeira, São Paulo 13484-350, Brazil; kellen.rodrigues.nut@gmail.com (K.C.C.R.); leandropereiram@hotmail.com (L.P.d.M.); rodrigopaulifca@gmail.com (J.R.P.); dcintra@yahoo.com (D.E.C.); eduardoropelle@gmail.com (E.R.R.)

**Keywords:** resistance exercise, autophagy, leucine, mammalian target of rapamycin (mTOR), liver, muscle

## Abstract

The present study verified the responses of proteins related to the autophagy pathway after 10 h of fast with resistance exercise and protein ingestion in skeletal muscle and liver samples. The rats were distributed into five experimental groups: control (CT; sedentary and without gavage after fast), exercise immediately (EXE-imm; after fast, rats were submitted to the resistance protocol and received water by gavage immediately after exercise), exercise after 1 h (EXE-1h; after fast, rats were submitted to the resistance protocol and received water by gavage 1 h after exercise), exercise and supplementation immediately after exercise (EXE/Suppl-imm; after fast, rats were submitted to the resistance protocol and received a mix of casein: whey protein 1:1 (*w*/*w*) by gavage immediately after exercise), exercise and supplementation 1 h after exercise (EXE/Suppl-1h; after fast, rats were submitted to the resistance protocol and received a mix of casein: whey protein 1:1 (*w*/*w*) by gavage 1 h after exercise). In summary, the current findings show that the combination of fasting, acute resistance exercise, and protein blend ingestion (immediately or 1 h after the exercise stimulus) increased the serum levels of leucine, insulin, and glucose, as well as the autophagy protein contents in skeletal muscle, but decreased other proteins related to the autophagic pathway in the liver. These results deserve further mechanistic investigations since athletes are combining fasting with physical exercise to enhance health and performance outcomes.

## 1. Introduction

Autophagy degrades macromolecules and organelles to recycle bioenergetics components [1,2], performing a fundamental role in cellular growth and development, organelles biogenesis, and balance between protein synthesis and degradation [3]. The autophagy pathway is activated at basal conditions and dramatically increases in stressful conditions like energy deficit and physical exercise [4]. The initiation process begins with the autophagic protein complex (ULK) formation, which includes the autophagic protein 1 (ATG1 or ULK1), ATG13, and focal adhesion kinase family interacting protein of 200 kD (FIP200 or ATG17). After that, a second complex is activated and contains the mammalian orthologue of yeast Atg6 (Beclin 1), phosphatidylinositol-3-kinases (PI3K), ATG14, p150, and Ambra1. In this stage, the Beclin 1 is disconnected to B cell lymphoma 2 (BCL-2), and this dissociation is responsible for the conduction of the complex to the phagophore membrane, starting the nucleation. The ATG7 and ATG3 proteins generate the lipidation of the microtubule-associated protein I (LC3 I), forming the LC3 II that is conjugated to a phosphatidylethanolamine generating the closure of phagophore in autophagosomes. The LC3 II also interacts with p62, the protein that marks who should be wrapped by autophagosomes and degraded by autophagy. In sequence, the fusion occurs with the lysosome to form the autolysosome for degradation [3,5,6]. 

Regular exercise has long been known to influence autophagy in rodents and humans [7,8]. Acute exercise requires a change in the cell and tissue integrity, such as an increase in mitochondrial activity, improving the generation of reactive oxygen species (ROS), leading to lipid peroxidation and protein carbonylation, and changing intracellular calcium homeostasis which results in the activation of intracellular proteases [9]. Altogether, these exercise factors influence cellular function, and autophagy has an important role in maintaining and restoring cell viability [9]. By the conversion of LC3I to LC3II and the presence of autophagosomes, Grumati et al. [10] showed that physical exercise activated autophagy in skeletal muscle. After ultra-endurance exercise, autophagy-related genes and proteins were upregulated in human’s vastus lateralis [11,12]. Activation of autophagy is related to exercise duration and/or intensity [9]. For instance, Schwalm et al. [13] demonstrated that the higher the exercise intensity, the higher the autophagy pathway. 

The increase of proteins related to the autophagy pathway in skeletal muscle after endurance exercise has been considered a clearance form of the contractile activity-induced metabolites [14], which may be the result of the attenuation of insulin/protein kinase B (Akt) signaling and intracellular energy [14]. Otherwise, in response to resistance exercise, some markers of autophagy decrease or remain unchanged in skeletal muscle since the mammalian target of rapamycin (mTOR) and insulin pathway activations are related to autophagy inhibition [5,15]. For instance, Fry et al. [16] demonstrated that after an acute resistance exercise, autophagy was reduced in the muscle of young and old adults. Also, Kwon et al. [17] verified that a six-week chronic resistance exercise protocol decreased autophagy markers in the flexor digitorum profundus muscle. Nutrient availability is also an important factor to consider [9]. Jamart et al. [18] showed that exercise performed in a fasted state increased LC3BII levels compared to the fed state. Consuming protein before, during, or after an acute exercise can stimulate muscle protein synthesis (MPS) [19,20,21]. Post-exercise protein feeding modifies skeletal muscle transcriptome responses to those supporting the endurance phenotype [21,22]. Essential amino acids (EAA) such as L-leucine can activate the mTOR pathway and inhibit autophagic flux, but their effects in skeletal muscle are not completely understood [14,23]. 

In the liver, the autophagy maintains a positive energy balance through the degradation of intracellular reserves of macromolecules such as proteins and lipids storages, which can be used as cellular fuel by the control of regulatory enzymes from the cellular metabolism such as glycolytic enzymes and preservation of mitochondrial homeostasis [24,25,26]. The hepatic autophagy activation occurs during starvation due to an increase in the circulating glucagon level as well as decreases in the circulating levels of insulin and amino acids [24,25,26].

Protein fractions such as whey protein (WP) and casein (CA) are found in milk. WP has a higher content of branched-chain amino acids (BCAA) and is considered a “fast” protein, while CA is considered a “slow” protein due to its structure that affects the absorption speed [27,28]. Although post-exercise protein feeding has been extensively used as a nutritional strategy by athletes [29,30], the combined effects of acute resistance exercise and protein ingestion after a fasting period on the autophagy pathway are not completely understood. Here, we investigate the responses of some autophagy markers in skeletal muscle and liver to the combination of acute resistance exercise with protein ingestion after a 10-h fasting period.

## 2. Material and Methods

### 2.1. Experimental Animals

Ten-week-old male Wistar rats from the Central Animal Facility of the Ribeirão Preto campus from the University of Sao Paulo (USP) were housed in polypropylene cages (two animals per cage) in a rack with controlled temperature (22 ± 2 °C) on a 12:12-h light-dark inverted cycle (light: 6 PM to 6 AM, dark: 6 AM to 6 PM) with food (Nuvilab^®^CR1; Sogorb Indústria e Comércio Ltda, São Paulo, SP, Brazil) and water provided ad libitum. The diet macronutrient composition was 63% carbohydrates, 26% proteins, and 11% lipids. All experimental procedures were performed according to the Brazilian College of Animal Experimentation (COBEA) and were approved by the Ethics Committee of the University of Sao Paulo (ID 2018.5.14.90.3). All applicable international, national, and/or institutional guidelines for the care and use of animals were followed.

The animals were distributed into five experimental groups: control (CT, *n* = 4; sedentary and without gavage after 10 h of fast), exercise immediately (EXE-imm, *n* = 4; after 10 h of fast, rats were submitted to the resistance protocol and received water by gavage immediately after exercise), exercise after 1 h (EXE-1h, *n* = 4; after 10 h of fast, rats were submitted to the resistance protocol and received water by gavage 1 h after exercise), exercise and supplementation immediately after exercise (EXE/Suppl-imm, *n* = 5; after 10 h of fast, rats were submitted to the resistance protocol and received a mix of casein: whey protein 1:1 (*w*/*w*) by gavage immediately after exercise), exercise and supplementation 1 h after exercise (EXE/Suppl-1h, *n* = 5; after 10 h of fast, rats were submitted to the resistance protocol and received a mix casein: whey protein 1:1 (*w*/*w*) by gavage one after exercise).

### 2.2. Acute Resistance Exercise Protocol and Supplementation

**Figure 1:**
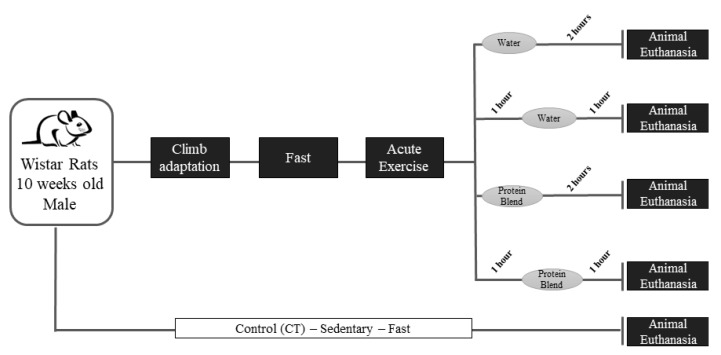
Experimental design timeline. Ten-week-old male Wistar rats were acclimatized to the exercise protocol (5 days of exercise adaptation and 2 days of rest). After one week, the animals fasted for 10 h and performed the acute resistance exercise protocol. Immediately or 1 h after the acute resistance exercise protocol, the animals received water or protein blend. The animals´ euthanasia for tissue extraction occurred at 2 h after the acute resistance exercise protocol.

The animals were acclimatized for five days (4 bouts per day) with the first three days without external load and the other two days with an external load corresponding to 50% of their body weight, which was attached to the tail. In the other two days of the first week, the animals remained at rest. The acute resistance exercise protocol was performed after a 10-h fast period. Each animal performed ten climbs with 75% of their body weight attached to the tail, with 2 min of rest between each climb. The acute resistance exercise protocol was based on the 6-week chronic resistance exercise protocol proposed by Kwon et al. [17], which used the intensity corresponding to 75% of body weight in weeks 3 and 4. In addition, this chronic protocol increased muscle hypertrophy, which was related to the potentization of anabolism and restriction of autophagy-induced catabolism. At the end of the acute exercise, the animals received water or protein blend by gavage immediately or 1 h after exercise, as summarized in Figure 1. The protein blend consisted of a mix of casein (50%) and whey protein (50%) (Nutratec^®^, Ribeirão Preto, SP, Brazil) with 3.1 g of protein per body weight diluted in 2.4 mL/100 g body weight [31]. Casein presented 121.95 g of protein/100 g and whey protein 133.35 g of protein/100 g. Table 1 shows the protein blend composition of amino acids in 100 g of the product label.

### 2.3. Skeletal Muscle and Liver Extraction

The rats were euthanized after 2 h of the acute exercise protocol with an intraperitoneal injection of xylazine (10 mg/kg of body weight) and ketamine (100 mg/kg of body weight). As soon as the anesthesia was confirmed by loss of pedal reflexes, their blood, mixed portion of gastrocnemius muscle, and liver samples were extracted and used for biochemical analysis and immunoblotting. All the gastrocnemius was extracted and weighed in analytical balance (Shimadzu^®^, Barueri, SP, Brazil).

### 2.4. Biochemical Analysis

After anesthesia and confirmation of loss of pedal reflexes, blood samples were collected by cardiac puncture to determine concentrations of the following biomarkers: insulin, glucose, triglycerides, and albumin. The serum was separated by centrifugation (1100× *g*) for 15 min at 4 °C and stored at −80 °C for the subsequent determinations. The commercially available conventional test kits (Labtest Diagnóstica S.A., Lagoa Santa, MG, Brazil) were used to measure glucose, triglycerides, and albumin (chemiluminescence). The serum insulin was measured by the ELISA kit (Crystal Chem®, Elk Grove Village, IL, USA) following the manufacturer’s instructions.

### 2.5. Leucine Dosage

Serum leucine concentration analysis was performed using a Shimadzu® high-pressure liquid chromatography (HPLC), model LC 10AD (Shimadzu Corporation, Tokyo, Japan), consisting of 2 continuous flow pumps for the mobile phase elution gradient. Samples of 10 μL of serum were added in 200 μL of methanol and placed in 1.5 mL tubes and centrifuged (Jouan Refrigerated Centrifuge, Model MR1812, Analytical Instruments Brokers LLC, Golden Valley, MN, USA) for 10 min at 1800 *g*. The supernatant was transferred to another 1.5 mL tube, and the methanol evaporated. After the total evaporation of the methanol, the dry serum was dissolved with 100 μL of the solution phase A. 10 μL of each sample were taken and placed in tubes suitable for analysis in the sampler (Sil 10A) with a capacity for 79 tubes. Then, 25 μL of the reaction product was injected into the C18 silica column, and the amino acids separated according to molecular weight and polarity and detected by the Shimadzu fluorescence detector model RF535, at 335 mm excitation and 455 mm emission [32].

#### Reverse Transcription-Quantitative Polymerase Chain Reaction (RTq-PCR)

Gastrocnemius and liver samples were collected and stored in RNAlater solution (Ambion, Foster City, CA, USA). All procedures were made under standard RNase-free conditions to avoid exogenous RNase contamination. Total RNA was extracted from ~50 mg of the gastrocnemius and liver using TRIzol® Reagent (Thermo Fischer Scientific, Waltham, MA, USA) according to the manufacturer´s instructions. Total RNA was measured by spectrophotometer at optical density 260, and quality was checked by the OD 260/280 ratio (BioDrop µLite, Biochrom, Holliston, MA, USA). cDNA was synthesized with 1000 ng of total RNA using High-Capacity cDNA Reverse Transcription Kit (Applied Biosystems, Foster City, CA, USA). Quantitative real-time PCR was performed using the StepOne Plus PCR System (Applied Biosystems, Foster City, CA, USA) to analyze the relative mRNA expression of *Sqstm1* (sequestosome 1/p62) (Forward: ACAGCCAGAGGAACAGATGG; Reverse: GTAGAGACTGGAGTTCACCTGTA).

The amplification reactions (10 µL final volume) were performed in duplicate with the following reagents: 5 µL 2 × Power Sybr Master Mix (Thermo Fisher Scientific, Wilmington, DE, USA), 1 uL primer forward, 1 µL primer reverse, 1 µL cDNA diluted in 1:10, and 2 µL of H_2_O. *Gapdh* (Forward AAGAGGGATGCTGCCCTTAC; Reverse: CGGGACGAGGAAACACTCTC) was used as a reference gene for the normalization of the data. Each amplification reaction occurred in the standard cycling in the following cycles: 10 min at 95 °C and a further 40 cycles with 15 s at 95 °C and 1 min at 60 °C. Relative quantification was calculated by the 2-ΔΔCT method using the Thermo Fisher Cloud Software, RQ version 3.7 (Life Technologies Corporation, Carlsbad, CA, USA).

### 2.6. Immunoblotting Technique

The immunoblotting technique was performed as previously described [33,34]. Proteins were denatured by boiling in Laemmli sample buffer containing 100 mM dithiothreitol, run on an SDS-PAGE gel, and transferred to nitrocellulose membranes (GE Healthcare, Hybond ECL, RPN303D, Chicago, IL, USA). Transfer efficiency to nitrocellulose membranes was verified by brief staining of the blots with Ponceau red stain. In sequence, the membranes were blocked with Tris-buffered saline (TBS) containing 5% bovine serum albumin and 0.1% Tween-20, for 1 h at room temperature. The antibodies used for immunoblotting overnight at 4 °C, in a dilution of 1:1000 were anti-ULK1 (SC-390908), phospho- mTOR (SC-293133), anti-p70S6K (SC-230), phospho-p70S6K (SC-11759), anti-GAPDH (SC-365062), and anti-alfa tubulin (SC-32293) from Santa Cruz (Santa Cruz Biotechnology, Dallas, TX, USA); phospho-ULK1 (OABF01248) from Aviva Systems Biology (Aviva Systems Biology Corporation, San Diego, CA, USA); anti-Beclin (66665-1-Ig), anti-mTOR (20657-1-AP), and anti-beta actin (66009-1-Ig) from Proteintech (Proteintech Group, Inc, Rosemont, IL, USA); anti-LC3B (3868S) from Cell Signaling Technology (Cell Signaling Technology, Danvers, MA, USA). 

After being washed with TBS containing 0.1% Tween-20, all membranes were incubated for 1 h at room temperature with the appropriate secondary antibody conjugated to horseradish peroxidase (dilution of 1:10,000; #7074s and #7076s) from Cell Signaling Technology (Cell Signaling Technology, Danvers, MA, USA). The specific immunoreactive bands were detected by chemiluminescence (GE Healthcare, ECL PlusWestern Blotting Detection System, RPN2132, Chicago, IL, USA). The images were acquired by the C-DiGit™Blot Scanner (LI-CORR, Lincoln, NE, USA) and quantified using the software Image Studio for C-DiGit Blot Scanner. Routine chemical reagents were purchased from Sigma Chemical Corporation (St. Louis, MO, USA). 

### 2.7. Statistical Analysis

Results are expressed as the mean ± standard error of the mean (SE). While the Shapiro–Wilk’s *W*-test was used to verify data normality, the Levene’s test was used to test the homogeneity of variances. When normality was confirmed, the one-way analysis of variance (ANOVA) followed by the post hoc of Bonferroni was used to examine the differences between the experimental groups. When normality was not confirmed, the Kruskal–Wallis test was used to examining the differences between the experimental groups. All statistical analyses were two-sided, and the significance level was set at *p* ≤ 0.05. Statistical analyses were performed using the software *SPSS* v.20.0 for Windows (IBM, Chicago, IL, USA).

## 3. Results

### 3.1. Body Weight, Gastrocnemius Weight, and Supplementation

Table 2 shows the body weight, gastrocnemius weight, and the amount (g) of casein and whey protein, which the experimental groups received. For Table 2, no significant differences were observed.

### 3.2. Insulin, Leucine, and Biochemical Analysis

**Figure 2:**
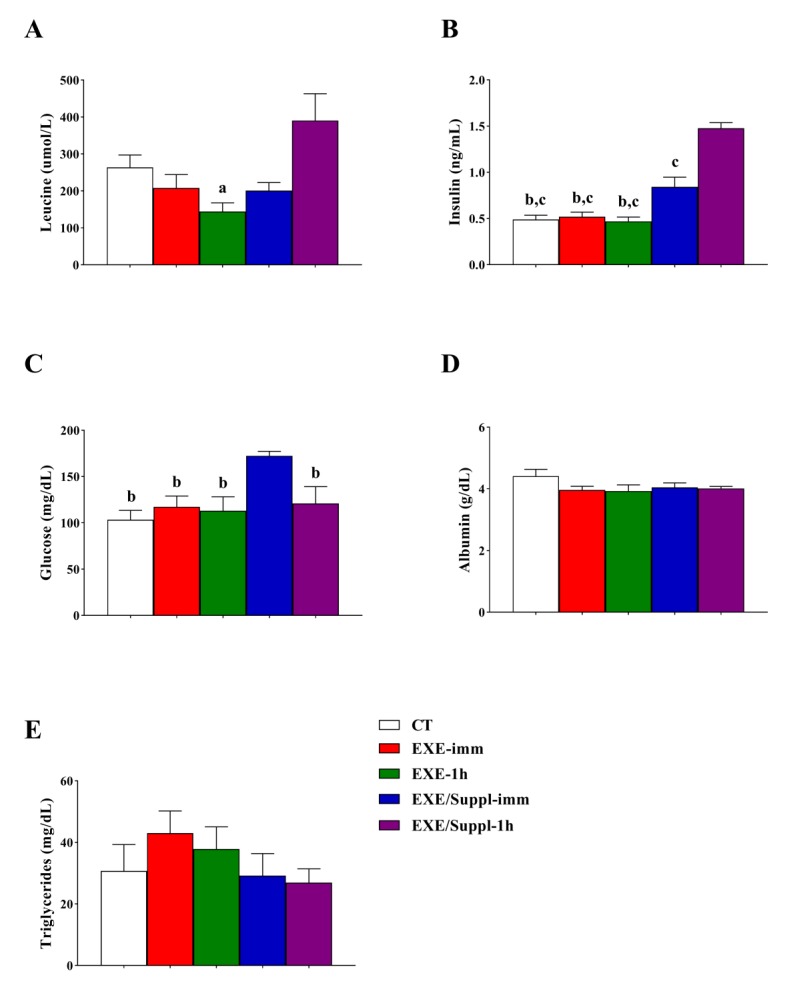
Serum levels of leucine (**A**), insulin (**B**), glucose (**C**), albumin (**D**), and triglycerides (**E**). Data correspond to the mean ± SE of *n* = 22 rats. CT: sedentary rats without gavage after 10 h of fast (*n* = 4). EXE-imm: after 10 h of fast, rats were submitted to the resistance exercise protocol and received water gavage immediately after exercise (*n* = 4). EXE-1h: after 10 h of fast, rats were submitted to the resistance exercise protocol and received water gavage 1 h after exercise (*n* = 4). EXE/Suppl-imm: after 10 h of fast, rats were submitted to the resistance exercise protocol and received a protein blend gavage immediately after exercise (*n* = 5). EXE/Suppl-1h: after 10 h of fast, rats were submitted to the resistance exercise protocol and received a protein blend gavage 1 h after exercise (*n* = 5). ^a^ *p* ≤ 0.05 versus EXE/Suppl-1h; ^b^ *p* ≤ 0.05 versus EXE/Suppl-imm; ^c^ *p* ≤ 0.001 versus EXE/Suppl-1h.

The serum leucine concentrations (µmol/L) (Figure 2A) were significantly higher for the EXE/Suppl-1h group compared to the EXE-1h group (*p* = 0.015). The insulin levels (Figure 2B) were higher for the EXE/Suppl-1h group (*p* ≤ 0.001) compared to the CT, EXE-imm, EXE-1h, and EXE/Suppl-imm groups. In addition, the insulin levels were higher for the EXE/Suppl-imm group compared to the CT (*p* = 0.035), EXE-imm (*p* = 0.038), and EXE-1h (*p* = 0.024) groups. The glucose levels (Figure 2C) were significantly higher for the EXE/Suppl-imm group compared to the CT (*p* = 0.005), EXE-imm (*p* = 0.034), EXE-1h (*p* = 0.019), and EXE/Suppl-1h (*p* = 0.05) groups. The albumin (Figure 2D) and triglycerides (Figure 2E) were not different between the experimental groups.

### 3.3. Gene and Proteins Related to the Autophagy Pathway in Gastrocnemius

**Figure 3:**
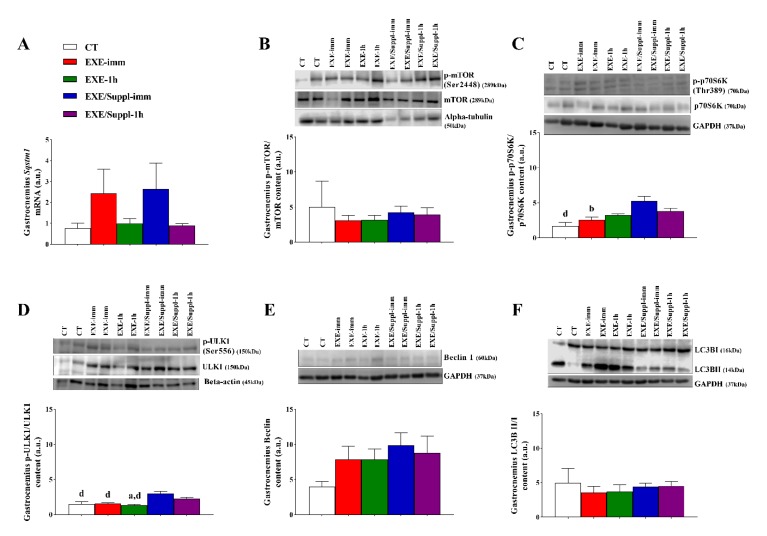
Gastrocnemius *Sqstm1* mRNA (**A**). Gastrocnemius protein levels (arbitrary units) of p-mTOR/mTOR (mammalian target of rapamycin) (**B**), p-p70S6K/p70S6K (**C**), p-ULK1/ULK1 (**D**), Beclin 1 (**E**), and LC3BII/LC3BI (**F**). Data correspond to the mean ± SE of *n* = 22 rats. When necessary, blots were stripped using Restore™ Plus Western Blot Stripping Buffer (Thermo Scientific, Waltham, MA, USA) and reprobed with antibodies that recognize the total form of a protein or control proteins. CT: sedentary rats without gavage after 10 h of fast (*n* = 4). EXE-imm: after 10 h of fast, rats were submitted to the resistance exercise protocol and received water gavage immediately after exercise (*n* = 4). EXE-1h: after 10 h of fast, rats were submitted to the resistance exercise protocol and received water gavage 1 h after exercise (*n* = 4). EXE/Suppl-imm: after 10 h of fast, rats were submitted to the resistance exercise protocol and received a protein blend gavage immediately after exercise (*n* = 5). EXE/Suppl-1h: after 10 h of fast, rats were submitted to the resistance exercise protocol and received a protein blend gavage 1 h after exercise (*n* = 5). ^a^ *p* ≤ 0.05 versus EXE/Suppl-1h; ^b^ *p* ≤ 0.05 versus EXE/Suppl-imm; ^d^ *p* ≤ 0.001 versus EXE/Suppl-imm.

Figure 3A shows that the *Sqstm1* mRNA was not different between the experimental groups. Figure 3B,E,F demonstrates that the p-mTOR/mTOR, Beclin, and LC3BII/LC3BI were not different between the experimental groups, respectively. Figure 3C shows that the p-p70S6K/p70S6K for the CT (*p* = 0.001) and EXE-imm (*p* = 0.007) groups were lower compared to the EXE/Suppl-imm group. Figure 3D shows that the p-ULK1/ULK1 for the CT (*p* = 0.001), EXE-imm (*p* = 0.001), and EXE-1h (*p* ≤ 0.001) groups were lower compared to the EXE/Suppl-imm group. In addition, the EXE-1h group had lower values of this protein (*p* = 0.046) compared to the EXE/Suppl-1h group.

### 3.4. Gene and Proteins Related to the Autophagy Pathway in the Liver

**Figure 4:**
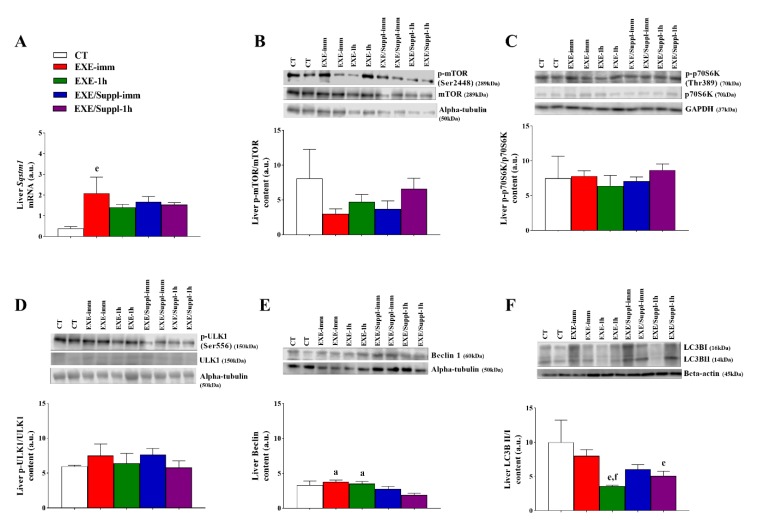
Liver *Sqstm1* mRNA (**A**). Liver protein levels (arbitrary units) of p-mTOR/mTOR (**B**), p-p70S6K/p70S6K (**C**), p-ULK1/ULK1 (**D**), Beclin 1 (**E**) and LC3BII/LC3BI (**F**). Data correspond to the mean ± SE of *n* = 22 rats. When necessary, blots were stripped using Restore™ Plus Western Blot Stripping Buffer (Thermo Scientific, Waltham, MA, USA) and reprobed with antibodies that recognize the total form of a protein or control proteins. CT: sedentary rats without gavage after 10 h of fast (*n* = 4). EXE-imm: after 10 h of fast, rats were submitted to the resistance exercise protocol and received water gavage immediately after exercise (*n* = 4). EXE-1h: after 10 h of fast, rats were submitted to the resistance exercise protocol and received water gavage 1 h after exercise (*n* = 4). EXE/Suppl-imm: after 10 h of fast, rats were submitted to the resistance exercise protocol and received a protein blend gavage immediately after exercise (*n* = 5). EXE/Suppl-1h: after 10-h of fast, rats were submitted to the resistance exercise protocol and received a protein blend gavage 1 h after exercise (*n* = 5). ^a^ *p* ≤ 0.05 versus EXE/Suppl-1h; ^e^ *p* ≤ 0.05 versus CT, ^f^ *p* ≤ 0.05 versus EXE-imm.

Figure 4A shows that the *Sqstm1* mRNA was higher for the EXE-imm group compared to the CT group (*p* = 0.023). Figure 4B–D demonstrates that the p-mTOR/mTOR, p-p70S6K/p70S6K, and p-ULK1/ULK1 were not different between the experimental groups, respectively. Figure 4E shows that the Beclin for the EXE-imm (*p* = 0.010) and EXE-1h (*p* = 0.034) groups were higher compared to the EXE/Suppl-1h group. Figure 4F shows that the LC3BII/I for the EXE-1h (*p* = 0.002) and EXE/Suppl-1h (*p* = 0.012) groups were lower compared to the CT group. In addition, the EXE-1h group had lower values of this protein (*p* = 0.042) compared to the EXE-imm group.

## 4. Discussion

The main findings of the present investigation were: (a) the serum leucine levels were statistically higher for the EXE/Suppl-1h group compared to the EXE-1h group; (b) the serum insulin levels were statistically higher for the EXE/Suppl-imm group compared to the CT, EXE-imm, and EXE-1h groups, as well as for the EXE/Suppl-1h group compared to all experimental groups; (c) the serum glucose levels were statistically higher for the EXE/Suppl-imm group compared to all experimental groups; (d) in the gastrocnemius, the p-p70S6K/p70S6K was statistically higher for the EXE/Suppl-imm group compared to the CT and EXE-imm groups, and the p-ULK1/ULK1 was statistically higher for the EXE/Suppl-imm group compared to the CT, EXE-imm, and EXE-1h groups; (e) in the liver, the Beclin was statistically lower for the EXE/Suppl-1h group compared to the EXE-imm and EXE-1h groups, and the LC3BII/I was statistically lower for the EXE/Suppl-1h group compared to the CT group. Altogether, these findings suggest that the combination of fasting, acute resistance exercise, and protein blend ingestion activated and inhibited different autophagy markers in gastrocnemius and liver, respectively.

Whey and casein proteins contain all essential amino acids [35]. Indeed, whey protein primarily contains leucine compared to other sources of protein, showing rapid digestion and increasing blood amino acid concentrations [36]. However, this effect returns to basal levels within 2–3 h [36]. When casein is ingested, the aminoacidemia is slower and prolonged [35]. The blend of whey protein and casein offers advantages over a single source of protein [35]. An interesting result of the present investigation was that the EXE/Suppl-1h group showed higher leucine values compared to the EXE-1h group, but without statistical difference compared to the EXE/imm group. According to Kato et al. [37], the physical exercise performed after the fasting session stimulates the oxidation of fats. The fact that the EXE-imm group did not show a significant difference in leucine levels compared to the EXE/Suppl-1h group suggests that the EXE-imm group probably had a greater fat contribution as an energetic substrate, reducing the participation of amino acids. Further studies should compare the contributions of energy from different substrate sources between EXE-imm and EXE-1h groups to test our hypothesis.

The EXE/Suppl-imm and EXE/Suppl-1h groups displayed increased levels of insulin compared to the other groups, while the EXE/Suppl-imm group also had elevated levels of glucose. Kanda et al. [27] visualized the peak of intramuscular and plasma leucine levels in male Sprague-Dawley rats that swam and received milk protein (whey protein and casein) at 60 min, but without significant alterations of plasma insulin concentrations. Further, Dijk et al. [31] observed elevated levels of intramuscular and plasma leucine in C57/BL6RJ mice at 25 months of age after 60, 75, and 90 min of leucine-enriched whey protein gavage, but with no changes in glucose concentrations measured at 60 and 75 min. Regarding the plasma insulin levels, the authors verified higher concentrations for the animals receiving only leucine compared to the fasted mice. In humans, the supplementation of leucine-enriched whey protein led to an insulin peak 30 min after intake, returning to baseline levels after 90 min [38], which corroborates with the current data showing that the EXE/Suppl-1h group displayed higher levels of insulin and leucine [31]. Esmarck et al. [39] visualized that a protein-carbohydrate-fat supplement was efficient in stimulating protein gain in senior men when ingested immediately after resistance exercise. Therefore, it is important to point out that the results found in rodents and humans are similar. 

A rich amino acid environment inhibits autophagy and activates the mTOR pathway [40]. mTOR complex 1 (C1) regulates the protein translation and ribosome biogenesis over phosphorylation of p70S6K and 4EBP1 positively. On the other hand, mTORC1 regulates the autophagy negatively through phosphorylation of ULK1 and ATG13 [41,42]. Although the p-mTOR/mTOR was unaffected by energy deficit (CT group) and energy deficit combined with exercise (EXE-imm and EXE-1h) in gastrocnemius, the p-p70S6K/p70S6K was higher for the EXE/Suppl-imm group compared to the CT and EXE-imm groups, which may be explained by the type of stimulus (resistance exercise) combined with elevated values of serum insulin. Interestingly, the p-ULK1/ULK1 was also upregulated for the EXE/Suppl-imm group. It is known that a mTORC1-independent pathway can regulate autophagy. For example, the AMP-activated protein kinase (AMPK) can inhibit mTOR and phosphorylate ULK1 or the transcription factor EB (TFEB), which stimulates autophagy-related factors [41]. In addition, Ogasawara et al. [43] injected AZD8055 (ATP-competitive mTOR kinase inhibitor) in the gastrocnemius of rats 1 h before the isometric muscle contraction and visualized an inhibition in the ULK1 phosphorylation, but without alteration of the LC3 expression.

Verdijk et al. [44] showed that protein supplementation immediately before and after exercise does not further enhance the increase in skeletal muscle mass after prolonged resistance-type exercise training in healthy older adults. Kwon et al. [17] observed that rats performing a long-term resistance exercise upregulated the p70S6K in the flexor digitorum profundus muscle, but also exhibited a significant reduction in autophagy, which was highlighted by a decrease of AMPK phosphorylation and LC3-II/LC3-I ratio. Kitaoka et al. [45] visualized that caloric restriction in rat muscles had no effects on the phosphorylation of mTOR signaling proteins rpS6 and ULK1. Furthermore, when inserting the resistance exercise, no effects were visualized on rpS6 and ULK1. In the investigation of Jamart et al. [18], mice performed a low-intensity running exercise (10 m/min for 90 min) in both dietary states (fed and fast). Corroborating our data, the levels of Beclin in gastrocnemius were unaffected by exercise, as well as both nutritional conditions [18]. Different from our study, the LC3BII/I ratio was higher in the fasted state with or without exercise compared to the fed state [18]. 

He et al. [46] showed that an interruption between B-cell lymphoma 2 (Bcl-2) and Beclin 1 was responsible for autophagy activation in response to exercise. In another investigation, an increase in LC3BII was observed in plantaris muscle after three days of fasting [47], which represents a longer time than the fasting period of the current investigation. In addition, rats from the present study were euthanized 2 h after the acute resistance exercise protocol, which may be considered an insufficient period to activate the analyzed proteins. It is important to highlight that proteins and their targets are phosphorylated at different periods [48]. 

In healthy humans, Møller et al. [49] examined skeletal muscle biopsies after 1 h of cycling at 50% of the maximal oxygen uptake during a 36-h fast period or continuous glucose infusion. These authors verified that physical exercise increased ULK1 phosphorylation at serine 555, which was positively correlated with AMPK phosphorylation at threonine 172, and decreased LC3BII/I. The fasting period elevated the protein levels of p62 and ULK1 but did not influence the exercise-induced ULK1 phosphorylation. Based on their findings, Møller et al. [49] concluded that 60 min of endurance exercise activates the autophagy pathway in human skeletal muscle regardless of the nutritional status. In the current investigation, we verified that the acute resistance exercise-induced ULK1 phosphorylation at serine 556 was higher only for the groups receiving the protein blend. More studies are necessary to elucidate the effects of different exercise modalities concerning duration, intensity, and cofactors such as nutritional and prior training status.

Regarding the *Sqstm1/p62* mRNA levels in the gastrocnemius, no difference was visualized among the experimental groups. In accordance, Kruse et al. [50] did not find significant differences for the *Sqstm1/p62* mRNA levels in skeletal muscle samples from patients with diabetes type II and their controls before, immediately after, and 3 h after a cycle ergometer exercise session for 60 min at an intensity of 70% of the maximal oxygen uptake. Jamart et al. [18] demonstrated an increase in the *Sqstm1/p62* mRNA in gastrocnemius of fasted mice submitted to running on a treadmill for 90 min at a speed of 10 m/min. The higher exercise volume of their protocol compared to ours (i.e., 90 min versus approximately 20 min) may have influenced the differences in *Sqstm1/p62* mRNA responses. Furthermore, Mônico-Neto et al. [51] verified that male Wistar rats, which were submitted to an eight-week resistance exercise protocol, did not present significant differences for the p62 protein levels in plantaris muscle. 

In the liver, autophagy occurs during starvation, increasing the circulating levels of glucagon and decreasing the circulating levels of insulin and amino acids, which are inhibitors of hepatic autophagy. Herein, the *Sqstm1* mRNA was higher for the EXE-imm group compared to the CT group. Hepatic autophagy activation during or after exercise stimulus is necessary for the recycling of cellular components and the elimination of damaged proteins [52]. In contrast to our data, Kwon et al. [53] verified that five days of moderate-intensity exercise decreased the protein levels of p62 in the liver of male C57BL/6 mice. Kristensen et al. [52] described that 60 min of running at 15 m/min and 10 degrees of inclination were not able to modulate the protein contents of p62 in the liver of wild-type mice. 

Insulin has an inhibitory effect upon intramuscular autophagy in mice [41], although amino acid availability can restrict the autophagic flux [54]. Naito et al. [41] reported that insulin and amino acids stimulate protein synthesis in the muscle, but only amino acids play this role in the liver since mainly amino acids regulate hepatic mTOR. Sato et al. [23] visualized that the mTOR signaling was recovered in rats fed with Lys-rich 10% casein diet but was decreased in the group fed with a standard 10% casein diet, which does not corroborate with the current findings. Dethlefsen et al. [55] observed an increase of hepatic LC3II/I ratio in animals with a high-fructose diet. Here, we observed that the EXE-1h and EXE/Suppl-1h groups had lower levels of LC3II/I ratio compared to the CT group. It is important to point out that the time of extraction probably played an essential role in the regulation of this protein. 

In contrast, Kristensen et al. [52] verified an increase in the hepatic LC3II/I ratio immediately after running exercise and fasting. Interestingly, the hepatic LC3II/I ratio was not modulated 2, 6, and 10 h after exercise. The Beclin protein was also downregulated for the EXE/Suppl-1h group compared to the EXE-imm and EXE-1h groups. Santos-Alves et al. [56] visualized an upregulation of Beclin and LC3II/I ratio in the liver of rats exercised on a treadmill for 12 weeks. In another study, animals receiving Doxorubicin, an effective anticancer agent, and training on a treadmill for 12 weeks did not modulate Beclin compared to the sedentary group [56]. To the best of our knowledge, this is the first investigation verifying the hepatic autophagy pathway in response to resistance exercise with and without protein blend supplementation. Therefore, further studies are necessary to better comprehend the association between different exercise modalities and protein ingestions on the regulation of autophagy adaptive processes in the liver.

**Figure 5:**
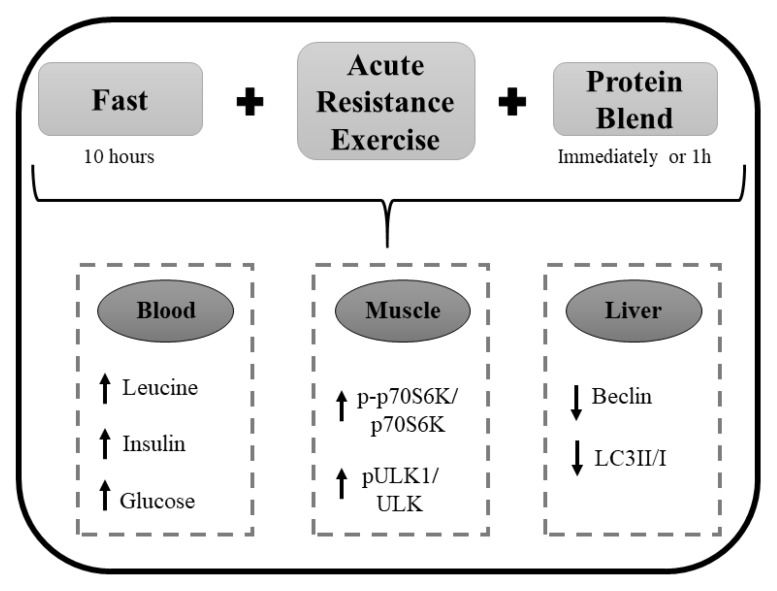
A schematic figure summarizing the findings of the present investigation.

In summary, the present data demonstrated that the association of a 10-h fasting period, acute resistance exercise, and protein blend supplementation (immediately or 1 h after the exercise stimulus) enhanced the serum concentrations of leucine, insulin, and glucose, as well as the autophagy protein contents in skeletal muscle, but diminished other proteins related to the autophagic pathway in the liver (Figure 5). These results are partially beneficial since autophagy is fundamental for the adaptation and remodeling of skeletal muscle in response to physical exercise [57]. On the other hand, the hepatic downregulation of Beclin and LC3II/I ratio observed in the current study deserves further mechanistic experiments since the deficiency of these autophagy markers is associated with an unhealthy liver [58,59]. It is interesting to point out that the same experimental design produced different responses according to the evaluated tissues. Thus, additional studies are necessary to elucidate the mechanism of autophagy after fast combined with resistance exercise and supplementation in specific tissues, once athletes mix training and fasting to achieve better results. 

## Figures and Tables

**Table 1:** Protein blend composition of amino acids (g/100 g).

Protein Blend	
Total (Casein + Whey Protein)	
Aspartic Acid	6.92 (2.59 + 4.33)
Glutamic Acid	11.96 (5.01 + 6.95)
Alanine	5.85 (3.91 + 1.94)
Arginine	5.12 (4.0 + 1.12)
Cystine	0.73 (0.00 + 0.73)
Phenylalanine	2.19 (0.88 + 1.31)
Glycine	8.48 (7.70 + 0.78)
Histidine	1.13 (0.35 + 0.78)
Isoleucine	2.96 (0.57 + 2.39)
Leucine	5.74 (1.36 + 4.38)
Lysine	5.58 (1.84 + 3.74)
Methionine	1.85 (0.39 +1.46)
Proline	6.68 (5.90 + 0.78)
Serine	2.81 (1.40 + 1.41)
Tyrosine	1.26 (0.09 + 1.17)
Threonine	3.52 (0.70 + 2.82)
Tryptophan	0.63 (0.00 + 0.63)
Valine	3.38 (1.05 + 2.33)

**Table 2:** Body weight, gastrocnemius weight, and supplementation.

Group	Body Weight (g)	Gastrocnemius Weight (g)	Supplementation (g)	
			Casein	Whey Protein
CT (*n* = 4)	417 ± 0.02	2.42 ± 0.16	---	----
EXE-imm (*n* = 4)	436 ± 0.03	2.88 ± 0.32	---	----
EXE-1h (*n* = 4)	427 ± 0.11	2.65 ± 0.23	----	----
EXE/Suppl-imm (*n* = 5)	449 ± 0.11	2.47 ± 0.08	0.85 ± 0.25	0.93 ± 0.05
EXE/Suppl-1h (*n* = 5)	472 ± 0.20	2.76 ± 0.14	0.94 ± 0.02	0.98 ± 0.05

CT: control; EXE-imm: exercise immediately; EXE-1h: exercise after 1 h; EXE/Suppl-imm: exercise and supplementation immediately after exercise; EXE/Suppl-1h: exercise and supplementation 1 h after exercise. Data are expressed in mean ± SE.
